# Protective Effects of *Labisia pumila* var. *alata* on Biochemical and Histopathological Alterations of Cardiac Muscle Cells in Isoproterenol-Induced Myocardial Infarction Rats

**DOI:** 10.3390/molecules20034746

**Published:** 2015-03-16

**Authors:** Roza Dianita, Ibrahim Jantan, Athirah Z. Amran, Juriyati Jalil

**Affiliations:** Drug and Herbal Research Center, Faculty of Pharmacy, Universiti Kebangsaan Malaysia, Jalan Raja Muda Abdul Aziz, Kuala Lumpur 50300, Malaysia; E-Mails: roza_f99@yahoo.com (R.D.); athirah.amran@yahoo.com (A.Z.A.); juriyati@pharmacy.ukm.my (J.J.)

**Keywords:** *Labisia pumila* var. *alata*, standardized extracts, cardioprotection, isoproterenol, myocardial infarction, alkylresorsinols

## Abstract

The study was designed to evaluate the cardioprotective effects of the standardized aqueous and 80% ethanol extracts of *Labisia pumila* var. *alata* (LPva) in isoproterenol (ISO)-induced myocardial infarction (MI) in rats. The extracts were administered to Wistar rats orally for 28 days with three doses (100, 200 and 400 mg/kg of body weight) prior to ISO (85 mg/kg)-induced MI in two doses on day 29 and 30. The sera and hearts were collected for biochemical and histopathological analysis after the rats were sacrificed 48 h after the first induction. The main components of the extracts, gallic acid, alkylresorcinols and flavonoids were identified and quantitatively analyzed in the extracts by using a validated reversed phase HPLC method. The extracts showed significant protective effects as pretreated rats showed a significant dose-dependent decrease (*p* < 0.05) in cardiac enzyme activities, *i.e.*, cardiac troponin I (cTnI), creatine kinase MB isoenzyme (CK-MB), lactate dehydrogenase (LDH), alanine transaminase (ALT) and aspartate transaminase (AST), when compared with ISO-control rats. There were significant rises (*p* < 0.05) in the activity of oxidase enzymes, *i.e.*, glutathione peroxide (GPx), catalase (CAT) and superoxide dismutase (SOD) of the pretreated rats, when compared with ISO-control group. Histopathological examination showed an improvement in membrane cell integrity in pre-treated rats compared to untreated rats. The major components of LPva extracts can be used as their biomarkers and contributed to the cardioprotective effects against ISO-induced MI rats.

## 1. Introduction

Myocardial infarction (MI) is an irreversible necrosis of tissue of a region of the myocardium caused by ischemia, which is a perfusion imbalance between demand and supply of blood to the heart *via* the coronary circulation. The evidence of MI can be identified by elevations of different proteins released into the blood by the damaged myocytes. These biomarkers include cardiac troponin T and I (cTnT and cTnI), creatine kinase MB isoenzyme (CK-MB), lactate dehydrogenase (LDH) and many others. Determination of cTnT or cTnI is a highly sensitive and specific analytical method for cardiac injury as these contractile proteins are released from myocardium during the myocardium tissue injury and disruption of myocyte membranes [1]. Isoproterenol (ISO), an adrenergic beta-receptor agonist, when administered in high doses is able to produce in an infarct-like myocardial lesion in rats which resemble the changes observed in human MI [2,3]. It was suggested that ISO may induce lyosomal hydrolase activities that may be responsible for the tissue damage and the infarcted heart [4]. Several mechanisms have been postulated to explain the cardiotoxic effects of ISO such as hypoxia-ischemia function, coronary insufficiency and oxidative stress [5]. The reactive oxygen species (ROS) generated during the MI especially after reperfusion would directly injure the cell membrane and cause cell death. They also stimulate the releasing of inflammatory cytokines which both ROS and cytokines play role on apoptosis and intracellular Ca^2+^ overload which eventually leading to necrotic stage [6].

Several natural products have been reported to have protective roles against ISO-induced MI in rats. Most of them possess antioxidant potentials which scavenge and stabilize the ROS leading to maintaining the MI biochemical parameters towards normal. These natural products were also shown to reduce the cardiac histopathology damage and normalize the electrocardiographic changes after ISO intoxication [5]. *Labisia pumila* var. *alata* (LPva) is used as a decoction to ease childbirth and during *post-partum* to help to firm and tone the vaginal and stomach muscles and to regain vitality. It is also used to regulate menstruation, relieve menstrual pain, anti-flatulence, anti-dysentery and it cures bone diseases [7]. Previous studies have identified several flavonoids and phenolics in the plant as the compounds responsible for its antioxidant activities [8,9]. It was also reported that the plant is rich in alkyl resorcinols and saponins [10]. Alkyl resorcinols were reported to be able to modulate the activities of enzymes involved in the formation of free radicals under physiological conditions such as lypooxygenase, cyclooxygenase and xanthine oxidase [11]. Recently we found that the extract of LPva exhibited antioxidant activity against human low-density lipoprotein peroxidation as well as antiplatelet aggregation [12]. The present study focused on evaluation of the cardioprotective effects of LPva on isoproterenol-induced MI in rats through analyses of the biochemical markers and histopathological parameters.

## 2. Results and Discussion

### 2.1. Identification of Chemical Markers of L. pumila Extracts

**Figure 1:**
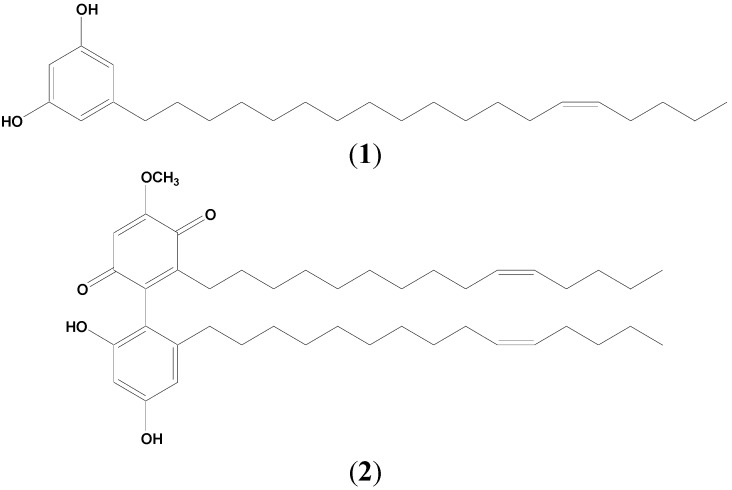
Chemical structures of 5-(Z-nonadec-14-enyl)resorcinol (**1**) and demethylbelamcandaquinone B (**2**).

**Figure 2:**
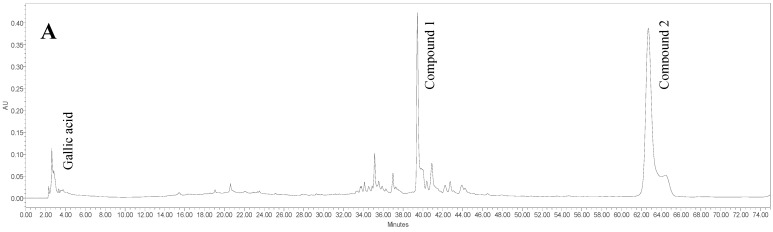

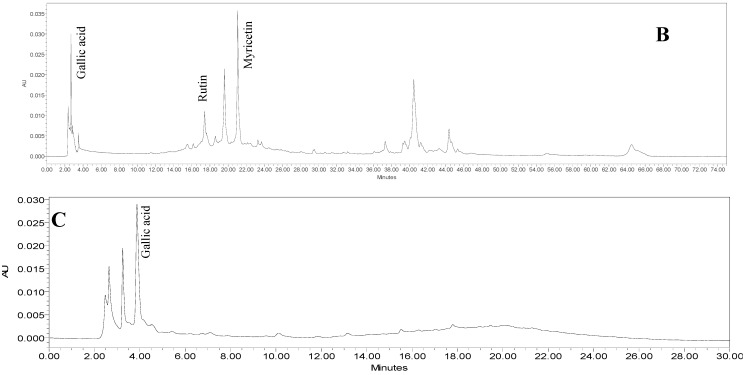
RP-HPLC chromatogram of the 80% ethanol extract of *Labisia pumila* var. *alata* at 280 nm (**A**) and 350 nm (**B**), for identification and quantification of 5-(*Z*-nonadec-14-enyl)resorcinol (compound **1**) and demethylbelamcandaquinone B (compound **2**), gallic acid, rutin and myricetin. RP-HPLC chromatogram of the aqueous extract (**C**) of *Labisia pumila* var. *alata* at 280 nm for identification and quantification of gallic acid.

Two major compounds*,* 5-(*Z*-nonadec-14-enyl)resorcinol (**1**) and demethylbelamcandaquinone B (**2**) (Figure 1), have been isolated from the n-hexane fraction of *L. pumila* var *alata* (LPva) extract via a series of chromatographic techniques. The identification of both compounds was carried out based on MS and NMR data and also by comparison with literature values [10,13]. The two compounds, along with a phenol (gallic acid) and two flavonoids (rutin and myricetin) were identified as the chemical markers of the standardized extracts of LPva by using a RP-HPLC method [9,10,14,15]. The method was developed from a previous method [14] with some modifications. Observation at 350 nm can detect gallic acid, rutin and myricetin peaks at RT 3.615 ± 0.031, 17.036 ± 0.340 and 20.615 ± 0.213 min, respectively, in both aqueous and 80% ethanol extracts of LPva. Another two peaks observed at 280 nm in the 80% ethanol extract were identified as compound **1** (RT 39.245 ± 0.173 min) and compound **2** (RT 61.714 ± 0.336 min), which were not detected in the aqueous extract (Figure 2).

### 2.2. Quantification of Aqueous and 80% Ethanol Extracts of L. pumila var. alata by Validated RP-HPLC Method

Amounts of the major compounds in the LPva extracts are shown in Table 1. Content of gallic acid in the aqueous extract (170.06 ± 0.01 µg/mL) was >2-fold higher than the ethanol extract (77.57 ± 0.11 µg/mL), whereas the amounts of myricetin and rutin were higher in the ethanol extract than in the aqueous one. Rutin was found three times higher than myricetin in the aqueous extract while both compounds were detected in almost equal amounts in the ethanol extract.

**Table 1:** Amount of major compounds found in *Labisia pumila* var. *alata* (µg/mL) obtained from HPLC quantification analysis.

Extract (10 mg/mL)	Amounts (µg/mL)				
	5-(*Z*-nonadec-14-enyl)resorcinol	Demethyl-belamcandaquinone B	Myricetin	Rutin	Gallic Acid
80%-EtOH extract	1006.87 ± 0.1	2793.61 ± 0.08	15.96 ± 0.16	16.89 ± 0.08	77.57 ± 0.11
Water extract	Not detected	Not detected	1.16 ± 0.01	3.51 ± 0.49	170.06 ± 0.01

Data is presented as mean ± SEM (n = 3).

The accuracy and the precision (intra- and inter-day) of the HPLC method were assessed using nine determinations (three concentrations/three replicates each). Table 2 shows that the current method has a good precision, as indicated by the small standard deviation values for both retention time and the responses of all five marker compounds. The current method also provided an acceptable degree of accuracy as indicated by its content uniformity within 80%–120% of the spiked concentration (Table 2). Five concentrations were used to establish calibration curves of the chosen standards in a range of 31.25–500 µg/mL. The calibration curve was made in triplicate for each standard. The LODs and LOQs of each standard are shown at Table 3.

**Table 2:** Accuracy (% recovery), intra- and inter-day precisions of the HPLC method.

Standard	Conc. (µg/mL)	Recovery * (%)	Intra-Day Precision **		Inter-Day Precision **	
			RT	Response	RT	Response
5-(*Z*-nonadec-14-enyl)resorcinol	31.25	104.89 ± 3.04	39.392 ± 0.02	52,392 ± 0.01	39.309 ± 0.06	52,690 ± 0.08
	62.5	103.74 ± 3.14	39.400 ± 0.04	106,716 ± 0.05	39.325 ± 0.06	106,589 ± 0.12
	125	107.93 ± 5.88	39.384 ± 0.04	241,753 ± 0.04	39.292 ± 0.08	236,970 ± 0.02
Demethylbelamcanda-quinone B	31.25	98.22 ± 3.67	61.752 ± 0.52	710,140 ± 0.11	61.584 ± 0.42	754,214 ± 0.03
	62.5	100.41 ± 3.77	61.537 ± 0.44	1,318,115 ±0.10	61.921 ± 0.08	1,394,224 ± 0.03
	125	100.36 ± 1.49	61.754 ± 0.28	2,495,414 ± 0.06	61.735 ± 0.30	2,539,039 ± 0.07
Gallic acid	31.25	94.60 ± 5.67	3.607 ± 0.01	46,449 ± 0.05	3.613 ± 0.01	45,190 ± 0.05
	62.5	97.07 ± 3.14	3.663 ± 0.01	81,207 ± 0.03	3.659 ± 0.02	82,606 ± 0.02
	125	98.22 ± 3.67	3.675 ± 0.05	169,362 ± 0.04	3.699 ± 0.03	165,185 ± 0.02
Myricetin	31.25	91.21 ± 2.66	20.590 ± 0.03	569,867 ± 0.05	20.619 ± 0.08	596,883 ± 0.04
	62.5	104.03 ± 2.76	20.551 ± 0.03	1,213,120 ±0.14	20.542 ± 0.11	1,227,027 ± 0.05
	125	99.15 ± 1.56	20.541 ± 0.04	2,132,241 ± 0.05	20.616 ± 0.20	2,159,026 ± 0.03
Rutin	31.25	99.81 ± 2.83	16.607 ± 0.23	722,316 ± 0.01	17.017 ± 0.35	788,152 ± 0.04
	62.5	93.44 ± 2.39	16.962 ± 0.18	1,712,277 ±0.01	17.007 ± 0.47	1,617,376 ± 0.06
	125	98.51 ± 3.21	17.208 ± 0.14	2,830,868 ± 0.01	17.089 ± 0.36	2,987,506 ± 0.11

Data is presented as: * mean ± SD (n = 3); ** mean ± SEM (n = 3).

**Table 3:** Regression parameter, LOD and LOQ of the HPLC method.

Standard	Conc Range (µg/mL)	Regression Equation	*R*^2^	LOD (ng/mL)	LOQ (ng/mL)
5-(*Z*-nonadec-14-enyl)resorcinol	31.25–500	y = 1672x + 20,149	0.998	0.69	2.08
Demethylbelamcandaquinone B	31.25–500	y = 16,378x + 45,378	0.999	0.89	2.70
Gallic acid	31.25–500	y = 1051.8x + 18,227	0.998	0.35	1.05
Myricetin	7.81–125	y = 22,051x − 274,315	0.995	0.03	0.08
Rutin	7.81–125	y = 24,591x + 21,313	0.999	0.14	0.43

### 2.3. Effects of L. pumila var alata Extracts on Serum Cardiac Troponin I (cTnI) Level

ISO-induced myocardial infarction in rats is one of the animal models widely used to evaluate the effectiveness of herbs or new drug entities. ISO acts directly on cardiac cardioprotective and vascular β-receptors resulting in hypoxia-ischemia conditions and eventually leading to necrosis of the myocardium, which are more prominent in the subendocardial tissue of the left ventricle [2]. Reperfusion of ischemic tissues results in the formation of toxic ROS such as superoxide anions, hydroxyl radicals, hypochlorous acid, hydrogen peroxides and nitric oxide-derived peroxinitrites. These ROS directly damage cellular membranes and stimulate releasing of leukocytes and inflammatory cytokines [16].

Troponin is a myocyte protein that regulates actin-myosin cross-bridge formation during the contraction-relaxation process in striated muscles. It consists of three different subunits: troponin T (TnT), troponin C (TnC) and troponin I (TnI). Both TnT and TnI have three different isoforms each of which is a structure unique to cardiac muscle [17]. A study showed that the content of cTnI and cTnT in skeletal muscle was below 0.6% that found in the heart [18], making troponin a specific biomarker in myocardiac infarction. It is noted that cTnI and cTnT are not detected in the blood under healthy conditions, as shown in the normal control serum (0.02 ± 0.01 ng/mL) in our study. Cardiac troponin elevations begin 2–4 h after onset of injury or infarction and persist for days [19,20].

**Figure 3:**
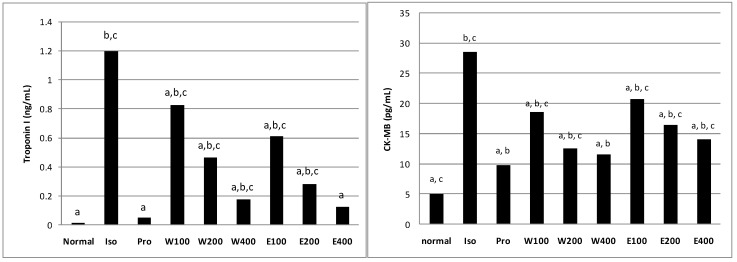
Effect of the aqueous and 80% ethanol extracts of *Labisia pumila* var. *alata* at different concentrations on serum troponin I (cTnI) and CK-MB isoenzyme in normal control and experimental myocardial infarction rats. ISO = isoproterenol, Pro = propranolol. ^a^ Significantly different to isoproterenol-induced (negative control) group at *p* < 0.05; ^b^ Significantly different to normal group at *p* < 0.05; ^c^ Significantly different to propranolol (positive control) group at *p* < 0.05.

Figure 3 shows a significant rise (*p* < 0.05) of cTnI level in the serum of MI rats (negative control) which was about 12-fold of the normal rats. Pre-treatment with both aqueous and 80% ethanol extracts of *L. pumila* successfully reduced (*p* < 0.05) the release of cTnI in a dose-dependent manner, indicating a protective effect against the injury due to ISO. At 400 mg/kg, the 80% ethanol extract showed cTnI level that was comparable (*p* < 0.05) to that of the normal group, as was shown too by the propranolol-treated group (Group III). Increased levels of serum cTnI reflected the relationship between cardiac lesions development and precede maximal lesion severity in acute models of MI [21]. Hence, low elevations of serum cTnI in both aqueous and 80% ethanol extracts of LPva (10–40 fold) when compared to ISO-induced rats (80-fold) indicated a protection given by LPva towards membrane cell integrity which eventually reduced the severity of cardiac injury.

### 2.4. Effects of L. pumila var alata Extracts on Other Cardiac Serum Marker Enzymes

The enzyme CK-MB is one of three CK isoenzymes. Unlike CK, which is found in many tissues of the body, CK-MB is present in a relatively high concentration in the myocardium (about 20% of the total myocardial CK), whereas only up to 2% can be found in healthy skeletal muscle. This fact makes CK-MB as specific biomarker to evaluate the evidence and severity of myocardial injury after cardiac troponin. Elevation of CK-MB occurs 4 to 9 h after the infarction, peaks at 24 h and returns to baseline at 48–72 h [22]. As shown in Figure 2, CK-MB levels in the normal group was considerably lower and more CK-MB was released into circulation during the cardiac injury, raising its serum level to nearly 6-fold in MI rats (*p* < 0.05). Pretreatment with the LPva extracts significantly (*p* < 0.05) reduced the release of this enzyme dose-dependently. At a dose of 400 mg/kg, water extract of LPva showed no significant different on serum CK-MB level compared to control positive, propranolol 10 mg/kg. It is suggested that pre-treatment with *L. pumila* extracts has successfully reduced the damage in the cardiac tissues which was revealed by lower CK-MB levels in experimental rats.

Other than cTnI and CK-MB, lactate dehydrogenase (LDH), aspartate aminotransferase (AST) and alanine aminotransferase (ALT) are amongst enzymes used to diagnose the necrotic cells. These enzymes are found within the cell cytoplasm where they catalyze anaerobic and aerobic metabolic reactions in cells. Once the cardiomyocytes were injured due to isoproterenol administration, this would be followed by disruption of associated cell membranes and these intracellular proteins were then released into the circulation, promptly increasing the serum levels of these enzymes during the acute phase of necrosis [23]. Although LDH, AST and ALT are not specific enzymes for MI due to their wide distribution in the body, they can be used as an early predictor of tissue damage [24]. Since isoproterenol induces myocyte necrosis even at low doses [25], it is less likely that the elevation of LDH, AST and ALT serum in this study were due to damage in heart tissue.

Table 4 shows a significant increase in the levels of all the other diagnostic marker enzymes (LDH, AST and ALT) in the MI rats (*p* < 0.05). Pre-treatment with LPva extracts prior to the challenge showed significant (*p* < 0.05) dose-dependently decreased levels of all those enzymes as compared to ISO-induced group (negative control). At a dose of 400 mg/kg, the 80% ethanol extract reduced the release of LDH to a level that was comparable to that of propranolol 10 mg/kg. The same effect was shown by 400 mg/kg of the aqueous extract on reducing the AST level. Hypothetically, LPva extracts significantly helped maintain the integrity of the membrane cells, thus restricting the leakage of these enzymes. The severity of the myocardial necrosis in experimental rats was reduced with an increase of the given dose of LPva, which is in accordance with the findings from previous studies [26,27,28].

### 2.5. Effect of L. pumila var alata Extracts on Serum and Cardiac Antioxidant System

During ischemia and reperfusion, oxygen-derived free radicals may form from several sources such as catecholamine degradation, mitochondrial dysfunction, leucocyte activation and xanthine oxidase. Metabolism of isoproterenol through monoamine oxidase system could generate hydrogen peroxide and hydroxyl radicals.

**Table 4:** Effects of aqueous and 80% ethanol extracts of *L. pumila* var. *alata* on cardiac marker enzymes and antioxidant system in rats.

Group	Serum Cardiac Marker Enzymes			Serum and Heart Antioxidant System					
	LDH	AST	ALT	SOD		CAT		GPx	
				Serum	Heart	Serum	Heart	Serum	Heart
Group I	127.83 ± 13.52 ^a,c^	51.40 ± 2.26 ^a^	21.10 ± 0.73 ^a^	290.37 ± 3.68 ^a,c^	15.05 ± 0.46 ^a,c^	296.45 ± 15.60 ^a,c^	19.13 ± 1.23 ^a,c^	81.69 ± 11.04 ^a,c^	340 ± 7.68 ^a,c^
Group II	215.11 ± 18.11 ^b,c^	92.44 ± 4.22 ^b,c^	55.91 ± 1.70 ^b,c^	217.91 ± 8.91 ^b,c^	9.30 ± 0.70 ^b,c^	63.63 ± 12.86 ^b,c^	9.37 ± 1.42 ^b,c^	15.52 ± 3.37 ^b,c^	38.14 ± 0.86 ^b,c^
Group III	157.10 ± 5.28 ^a,b^	56.48 ± 0.67 ^a^	18.46 ± 0.96 ^a^	282.33 ± 1.42 ^a,b^	13.06 ± 0.32 ^a,b^	240.33 ± 16.89 ^a,b^	15.06 ± 0.28 ^a,b^	58.05 ± 5.78 ^a,b^	218.52 ± 20.13 ^a,b^
Group IV	190.36 ± 1.08 ^a,b,c^	71.14 ± 0.83 ^a,b,c^	35.98 ± 0.49 ^a,b,c^	238.42 ± 4.50 ^a,b,c^	10.06 ± 0.12 ^a,b,c^	131.38 ± 2.99 ^a,b,c^	13.71 ± 0.13 ^a,b,c^	22.81 ± 2.21 ^b,c^	52.85 ± 4.93 ^b,c^
Group V	185.44 ± 1.36 ^a,b,c^	64.75 ± 1.52 ^a,b,c^	28.22 ± 0.35 ^a,b^	250.86 ± 4.11 ^a,b,c^	10.62 ± 0.30 ^a,b,c^	176.41 ± 5.67 ^a,b,c^	14.49 ± 0.10 ^a,b,c^	27.65 ± 0.40 ^a,b,c^	69.66 ± 4.33 ^a,b,c^
Group VI	181.04 ± 1.10 ^a,b,c^	59.01 ± 1.51 ^a,b^	26.92 ± 1.26 ^a^	272.16 ± 1.58 ^a,b,c^	12.12 ± 0.04 ^a,b,c^	201.65 ± 9.10 ^a,b,c^	14.78 ± 0.13 ^a,b,c^	30.22 ± 1.17 ^a,b,c^	94.76 ± 1.86 ^a,b,c^
Group VII	178.10 ± 8.51 ^a,b,c^	84.57 ± 2.42 ^a,b,c^	47.21 ± 1.50 ^a^	260.25 ± 1.70 ^a,b,c^	11.11 ± 0.14 ^a,b,c^	94.78 ± 8.37 ^a,b,c^	13.13 ± 0.15 ^a,b,c^	26.14 ± 0.63 ^a,b,c^	62.59 ± 3.02 ^a,b,c^
Group VIII	173.99 ± 1.42 ^a,b,c^	75.57 ± 2.42 ^a,b,c^	42.62 ± 0.30 ^a^	266.50 ± 2.19 ^a,b,c^	11.81 ± 0.21 ^a,b,c^	119.57 ± 4.08 ^a,b,c^	13.46 ± 0.09 ^a,b,c^	28.63 ± 0.32 ^a,b,c^	85.83 ± 1.28 ^a,b,c^
Group IX	167.96 ± 2.14 ^a,b^	68.23 ± 0.76 ^a,b,c^	32.89 ± 1.90 ^a^	277.81 ± 1.15 ^a,b^	12.45 ± 0.16 ^a,b^	150.20 ± 10.56 ^a,b,c^	14.18 ± 0.29 ^a,b,c^	41.14 ± 4.92 ^a,b,c^	121.43 ± 10.93 ^a,b,c^

Data is presented as mean ± SD (n = 6). ^a^ Significantly different to isoproterenol-induced (negative control) group at *p* < 0.05; ^b^ Significantly different to normal group at *p* < 0.05; ^c^ Significantly different to propranolol (positive control) group at *p* < 0.05. Activity is expressed as U/L for serum LDH and AST; IU/L for serum ALT; U/mL for serum SOD, CAT and GPx; U/mg protein for myocardium SOD, CAT and GPx. LDH = Lactate dehydrogenase; AST = Aspartate transaminase; ALT = Alanine transaminase; SOD = Superoxide dismutase; CAT = Catalase; GPx = Glutathione peroxidase.

Reperfusion of ischemic myocardium which occurs in repeated doses of isoproterenol may contribute to oxygen-derived free radicals which can induce irreversible myocardial damage [29,30]. Normally, endogenous antioxidants such as glutathione system, catalase and superdioxide enzymes will enzymatically inactivate and regulate the free radicals in effort to protect the cardiac from further damage. Our study revealed that administration of two-repeated high dose of isoproterenol significantly decreased SOD, GPx and catalase levels which are consistent with findings in previous reports [31]. As shown in Table 4, administration of isoproterenol caused a significant (*p* < 0.05) decrease in GPx, CAT and SOD levels as compared to normal group. Beta-blocker propranolol had significantly (*p* < 0.05) reduced oxidative stress in injured cardiac while both water and 80% ethanol extracts of LPva provided significant (*p* < 0.05) defense towards oxidative stress in a dose-dependent manner.

In our observations, there was progressive decrease of GPx and catalase levels, which appeared superior to SOD in protecting the cells from ROS-mediated damage which is similar to a previous study [32]. Glutathione peroxidase (GPx), a selenium-dependent cytoplasmic enzyme, is one of the major enzymes involved in protecting the cells against peroxidation, particularly in the heart muscle. GPx catalyzes H_2_O_2_ into H_2_O and O_2_ [33]. Similarly, catalase acts enzymatically to inactivate ROS using either iron or manganese cofactor. SOD levels were observed to slightly decrease in ISO-induced rats as it is located within mitochondria which released first into cytoplasm and afterwards into bloodstream [34]. It was suggested that SOD continue to be released from injured myocardiac for a long time. The higher levels of GPx, SOD and catalase in rats pre-treated with LPva as compared to ISO-induced control rats indicated its protective effect against ROS during the myocardial injury.

### 2.6. Effects of L. pumila var alata on Cardiac Histopathology

**Figure 4:**
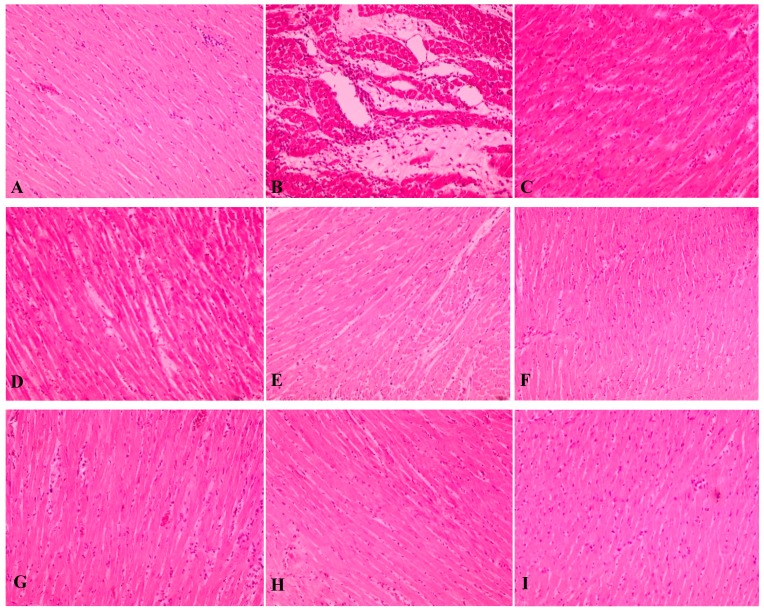
Effect of the aqueous (AqELP) and 80% ethanol (EtELP) extracts of *Labisia pumila* var. *alata* on histopathological changes, (**A**) control (normal), (**B**) ISO (negative control), (**C**) propranolol 10 mg/kg (positive control), (**D**): AqELP 100 mg/kg, (**E**) AqELP 200 mg/kg, (**F**) AqELP 400 mg/kg, (**G**): EtELP 100 mg/kg, (**H**): EtELP 200 mg/kg and (**I**): EtELP 400 mg/kg. Heart tissues (4 µm thickness) were stained with hematoxylin and eosin and visualized under light microscope at ×10 magnification.

Hispathological observations on cardiac tissues of control normal rats showed clear cell membrane integrity, normal myofibrillar structure with striations, appearance of branched and continuity with adjacent myofibrils. No evidence of inflammatory cell infiltration, edema or inflammation itself (Figure 4). However, the tissues of ISO-induced rats showed loss or blurring of striations following the necrosis of myofibrillar tissue. Degenerated myofibrils lost their nuclei due to infiltration of neutrophil granulocytes and macrophages causing an interstitial edema (Figure 4).

Pretreatment either with the aqueous and 80% ethanol extracts of LPva (100, 200 and 400 mg/kg, each, respectively) reduced the degree of necrosis and edema with obvious reduced infiltration of inflammatory cells. The improvements were observed to be in line with the given doses. At higher dose, both aqueous and 80% ethanol extracts show better protections to the myofibrillar as compared to rats given a lower dose. The same evidences were observed in tissues of the propranolol group (Figure 4). The histopathological findings revealed the evidences of severe myofibrillar degeneration with extensive neutrophil infiltration and interstitial edema in tissues of ISO-induced control group. Pre-treatment with LPva (100 mg/kg) reduced the severity of myocardial necrosis, which was indicated by mild neutrophil infiltration and edema in representative tissues. At the highest dose (400 mg/kg), the tissue was shown to have clear integrity, near to normal tissue histology, with slight evidence of focal necrosis and inflammatory cell infiltration. The membrane cell integrity protection given by LPva was slightly better compared to the control positive, propranolol. This protection can be postulated to be due to the ability of LPva to stabilize ROS during MI-phase, thus protecting myocardial membranes from lipid peroxidation and maintaining their permeability and integrity [35].

## 3. Experimental Section

### 3.1. General Information

The chemicals used in this study were of analytical grade. Absolute ethanol, formalin, toluene, xylene and silica gel for chromatography and other solvents for extraction, and histopathology were purchased from Merck Millipore (Darmstadt, Germany). Myricetin (99.0% purity), rutin (99.0% purity), gallic acid (99.0% purity), isoproterenol (ISO) (99.0% purity), propranolol (99.0% purity), Tween^®^ 20, Tris base, sterile-saline solution, hematoxyline, eosin, DPX for mounting were purchased from Sigma Chemical (St Louis, MO, USA). Methanol, acetonitrile, and trifluoroacetic acid of HPLC grade were purchased from Fisher Scientific (Loughborough, UK). Solvents and silica gel for isolation work were of analytical grade, was obtained from Merck Millipore (Darmstadt, Germany).

### 3.2. Sample Collection and Extracts Preparation

The whole plant of *Labisia pumila* var. *alata* was collected from Perak (Malaysia) between February and June 2012 and was identified by a botanist from the Institute of Bioscience, Universiti Putra Malaysia (UPM). The voucher specimen was deposited at the herbarium of Universiti Kebangsaan Malaysia (voucher no. UKMB 30010). No specific permissions were required for the collection of plant samples. The collection of plant samples did not involve endangered or protected species and the study was carried out at the Drug and Herbal Research Centre, Faculty of Pharmacy, Universiti Kebangsaan Malaysia, 50300 Kuala Lumpur, Malaysia.

A water extract of LPva was prepared by refluxing dried ground whole plant (1 kg) of LPva with 10 L of distilled water for 3 h. The extracts were filtered through Whatman^®^ No.1 filter paper (Sigma-Aldrich, St. Louis, MO, USA), pooled and subjected to freeze-drying to obtain a powdered extract. This crude extract was kept at 4 °C until further use. An 80% EtOH extract of LPva was obtained by macerating the dried ground whole plant (1 kg) of LPva with 80% ethanol (3 × 3 L) at room temperature for 3 days. The extracts were filtered, pooled and subjected to rotary evaporation to remove the residual solvent. The solvent-free extract was then freeze-dried to obtain a gummy-like crude extract. This crude extract was kept at 4 °C until further use.

### 3.3. Isolation Work

The dried powder of whole plant of LPva (2 kg) was soaked in *n*-hexane at the ratio of 1:10 (w/v) for 3 × 3 days; each time with 9 L of fresh solvent. After filtration, the filtrate was pooled and the residual solvent was evaporated under vacuum to obtain a crude extract (35.3 g). Part of the extract (20 g) was subjected to vacuum liquid chromatography (VLC) on silica gel type H (10–40 µm, 7 cm × 30 cm) and eluted with a gradient system of *n*-hexane: EtOAc to yield eight sub-fractions. Sub-fraction H6 (3.56 g) was subjected to repeated silica gel column (40–63 µm, 3 cm × 60 cm) with a gradient system of hexane–EtOAc (10:0–0:10, v/v) as mobile phase to yield nine sub-fractions. Sub-fraction H6F (632.9 mg) was further purified using silica gel with a gradient system of CHCl_3_–EtOAc (10:0–0:10, v/v) to yield 5-(*Z*-nonadec-14-enyl)resorcinol (**1**, 374.8 mg). Part of sub-fraction H6H (2.8596 g) was further purified on silica gel with CH_2_Cl_2_–EtOAc in a gradient polarity of 10:0–0:10, v/v, to yield demethylbelamcandaquinone B (**2**, 2.6433 g). The isolated compounds were identified based on their MS and NMR data and comparison with literature values [10,13]. Purity of the compounds was >98%, based on their physicochemical properties, NMR and ESI-MS data.

*5-(Z-nonadec-14-enyl)resorcinol* (**1**): Reddish oil; positive HRESI-MS *m/z* 375.2493 [M+H]^+^, calcd for C_25_H_42_O_2_); ^1^H-NMR (500 MHz, CDCl_3_) δ_ppm_ 1.05 (3H, *dd*, Me-19'), 1.35–1.53 (24H, m, H-2'-H-12', H-17'), 1.65 (2H, *m*, H-18'), 2.1 (4H, *m*, H-13', H-16'), 2.55 (2H, *dd*, H-1'), 5.51 (2H, *m*, *J* = 10 Hz, H-14', H-15'), 6.29 (1H, *s*, H-2), 6.38 (2H, *d*, *J* = 5 Hz, H-4, H-6). ^13^C-NMR (125 MHz, CDCl_3_) δ _ppm_ 156.08 (C-1, C-3), 146.46 (C-5), 129.87 (C-14', C15'), 108.29 (C-4, C-6), 100.35 (C-2), 35.92 (C-1'), 32.00 (C-2'), 14.02 (C-19').

*Demethylbelamcandaquinone B* (**2**): Red waxed-like solid; positive HRESI-MS m/z 663.4806 [M+H]^+^, calcd for C_43_H_66_O_5_); ^1^H-NMR (500 MHz, CDCl_3_) δ_ppm_ 1.02 (6H, t, Me-21, Me-21'), 1.25–1.60 (remaining CH_2_), 2.14 (8H, overlapped, *m*, H-15, H-18, H-15', H-18'), 2.29 (1H, *m*, H-7*_ax_*), 2.35 (2H, *m*, H-7'), 2.43 (1H, *m*, H-7*_eq_*), 3.96 (3H, *s*, OMe-1), 5.46 (4H, *m*, H-16, H-17, H-16', H-17'), 6.10 (1H, *s*, H-6), 6.29 (1H, *s*, H-5'), 6.31 (1H, *s*, H-3'), 7.06 (1H, *s*, OH), 7.26 (1H, *s*, OH). ^13^C-NMR (125 MHz, CDCl_3_) δ_ppm_ 188.59 (C-5), 182.15 (C-2), 159.08 (C-1), 156.54 (C-4'), 153.80 (C-6'), 146.99 (C-3), 142.96 (C-2'), 141.07 (C-4), 129.79 (C-16, C-17, C-16', C-16'), 112.0 (C-1'), 108.15 (C-3'), 107.25 (C-6), 100.93 (C-5'), 56.28 (OMe), 33.50 (C-7'), 31.94 (C-19, C-19'), 28.96–31.76 (remaining CH_2_), 26.89 (C-7, C-115, C-15', C-18, C-18'), 27.19 (C-14, C-14'), 22.31 (C-20, C-20'), 13.98 (C-21, C-21').

### 3.4. Quantitative Determination of the Major Components of Plant Extracts by HPLC

Sample solutions were prepared by dissolving 10 mg of the 80% EtOH extract of LPva with 1 mL of MeOH or 10 mg of water extract of LPva with 1 mL of MeOH–H_2_O (1:1, v/v) to give a concentration of 10 mg/mL. These stock solutions were then sonicated for 15 min and filtered through a 0.45 µm Millex PTFE membrane (Millipore, Maidstone, Kent, UK). Solutions for reference standards (myricetin, rutin, gallic acid, 5-(*Z*-nonadec-14-enyl)resorcinol and demethylbelamcandaquinone B) were prepared at a concentration of 1 mg/mL as above and further diluted into a series of two fold dilutions (3.81–500 µg/mL). These solutions were kept at −20 °C prior to analysis. HPLC analysis was performed on Waters 2535 Quartenary Gradient Module with Photodiode Array Detector (Waters 2998) with detection over the 210 to 350 nm wavelength range and a Waters Prep Degassser, using an XBridge™ C18 (4.6 mm × 250 mm, 5 µm) analytical column (Waters, Milford, MA, USA) The injection volume was 20 µL for sample extracts and 10 µL for reference standards. Data acquisition was performed with the Empower3 software.

The mobile phase consisted of solvent A (acetonitrile) and solvent B (0.1% orthosphosphoric acid). The initial composition was 10% solvent A and 90% solvent B followed by increasing solvent A to 100% in 30 min (curve 7) and maintained 100% A until 75 min. The detection was measured at 280 and 350 nm with a flow rate at 1 mL/min. The column was equilibrated with the initial composition for 15 min prior to sample injection and was washed with 100% methanol for 5 min in between sample injections. The identification of each compound was carried out by comparing the retention times and UV-Vis spectra of the peaks with those obtained by the injection of the standards. The calibration curves were plotted with five concentrations each of the standard solution of compounds *versus* the areas under the peaks. The standard curves equations obtained from each compounds were used to quantify the compounds in the extracts. The identification of each compound was carried out by comparing the retention times and UV-Vis spectra of the peaks with those obtained by the injection of the standards. The calibration curves were plotted with five concentrations each of the standard solution of compounds *versus* the areas under the peaks. The standard curves equations obtained from each compounds were used to quantify the compounds in the extracts.

### 3.5. Validation Procedures for HPLC Analysis

The HPLC method was validated according to the ICH guidelines for the validation of analytical procedures [36]. The validation included accuracy, precision, linearity, detection limit (LOD) and quantitation limit (LOQ). The accuracy was reported as percent recovery and assessed by spiking known reference(s) at certain amount to the blank sample over nine determinations (three concentrations/three replicates). The precision was investigated at two levels: repeatability (the precision under the same method protocol over a short interval of time, or intra-assay precision) and intermediate precision (evaluation of method performance on different days, or inter-day precision). Separately, one concentration of extracts (10 mg/mL) and reference compounds (31.25, 62.5, and 125 µg/mL) were injected three times for each concentration in one day and on three different days. The calibration curves were established by using 5-(*Z*-nonadec-14-enyl)resorcinol, dimethyl-belamcandaquinone B, gallic acid, myricetin and rutin as external standards. Six dilutions of each standard (3.91–500 µg/mL) were used in triplicate and the curve was constructed by plotting the corresponding peak areas (also known as responses) against concentrations of the injected standards. The linearity was evaluated by using regression parameter from calibration curve and correlation coefficient (*R*^2^), calculated from the calibration curves. LOD and LOQ were calculated from the RSD (residual standard deviation) and slope (*S*) of the calibration curves using equations:

LOD = 3.3 × (RSD/*S*) and LOQ = 10 × (RSD/*S*).

### 3.6. In Vivo Experimental Design and Protocol

Male Wistar rats (150–200 g) were purchased from a local supplier (Perniagaan Usaha Cahaya, Kuala Lumpur, Malaysia). The animals were housed at 25 ± 2 °C in a well-ventilated animal house under 12/12 h light and dark cycle. Animals were maintained under standard condition (humidity 60% ± 10%), fed with conventional diet and water provided *ad libitum*. There was no significant difference in the body weights of the treated rats when compared with control at the beginning of the study period. This study was carried out following an approval from the Universiti Kebangsaan Malaysia Animal Ethics Committee (FF/2012/IBRAHIM/23-MAY/433-MAY-2012-SEPTEMBER-2013).

Rats were divided into nine groups of six rats each.

Group I normal-control group.
Group II ISO-control group (received 85 mg/kg of ISO, s.c. on 29th and 30th day).
Group III rats treated with 10 mg/kg of propranolol, orally, for 28 days and received 85 mg/kg ISO, s.c. on 29th and 30th day.
Group IV rats treated with 100 mg/kg of LPva water extract orally, for 28 days and received 85 mg/kg of ISO, s.c. on 29th and 30th day.
Group V rats treated with 200 mg/kg of LPva water extract, orally, for 28 days and received 85 mg/kg of ISO, s.c. on 29th and 30th day.
Group VI rats treated with 400 mg/kg of LPva water extract, orally, for 28 days and received 85 mg/kg of ISO, s.c. on 29th and 30th day.
Group VII rats treated with 100 mg/kg of LPva 80% ethanol extract, orally, for 28 days and received 85 mg/kg of ISO, s.c. on 29th and 30th day.
Group VIII rats treated with 200 mg/kg of LPva 80% ethanol extract, orally, for 28 days and received 85 mg/kg of ISO, s.c. on 29th and 30th day.
Group IX rats treated with 400 mg/kg of LPva 80% ethanol extract, orally, for 28 days and received 85 mg/kg of ISO, s.c. on 29th and 30th day.

Samples (100 mg/mL) and propranolol (5 mg/mL) were individually dispersed homogenously in 2% Tween 20. Isoproterenol (10 mg/mL) was dissolved in water for injection and injected subcutaneously (s.c.) to the rats at interval of 24 h for 2 consecutive days. At 31st day, 48 h after the first induced, rats were sacrificed under mild diethyl ether anesthesia. Animals were made to fast 12 h before sacrificing. Blood was collected via abdominal aorta and allowed to clot for 1 h at room temperature. Serum was separated by centrifugation at 4000 rpm for 20 min and kept at −80 °C for further biochemical analysis. Heart tissue was excised immediately and rinsed with cold saline solution. The cardiac apex was dissected out and fixed in 10% formalin for histopathological examination. The remaining part was immediately homogenized in cold 0.1 M Tris-HCl buffer (pH 7.4, 1:10 w/v). The homogenate was then centrifuged at 13,000 rpm for 10 min at 4 °C and the supernatant was stored at −80 °C for further biochemical analysis.

### 3.7. Estimation of Cardiac Troponin I (cTn1)

The serum level of cardiac troponin I (cTnI) was estimated by using enzyme-linked immunosorbent assay kit (Abnova, Taoyuan, Taiwan).

### 3.8. Assay of Cardiac Marker Enzymes

The levels of serum creatine kinase-MB isoenzyme (CK-MB) were determined by using enzyme-linked immunosorbent assay kit (Cloud-Clone, Houston, TX, USA). Meanwhile, activities of other cardiac marker enzymes were measured by using colorimetric commercial standard kits including lactate dehydrogenase, LDH (BioVision, Milpitas, CA, USA), alanine transaminase, ALT (Cayman Chemical, Ann Arbor, Michigan, USA) and aspartate transaminase, AST (BioAssay System, Hayward, CA, USA).

### 3.9. Antioxidant System Assays

Analysis of antioxidants was performed by measuring the glutathione peroxide (GPx), catalase (CAT) and superoxide dismutase (SOD) levels in serum and myocardium homogenate using commercial standard assay kits (Cayman Chemical).

### 3.10. Histopathological Examination

The formalin-fixed tissues were dehydrated in a series of graded alcohol and toluene followed by embedding in paraffin wax. The tissue was then cut into 4 µm thickness by using microtome and stained with hematoxyline and eosin. The tissue examination was performed under light microscope and photographed by using a BX41 digital camera (Olympus, Tokyo, Japan).

### 3.11. Statistical Analysis

Data are presented as mean ± SD. Results were analyzed statistically by using IBM SPSS Statistics 21. One-way ANOVA and Tukey’s test were carried out to statistically compare the data among the groups. The *p*-values less than 0.05 were considered as statistically significant.

## 4. Conclusions

HPLC analysis of the 80% ethanol extract of LPva revealed the presence of two alkylresorcinols, (5-(Z-nonadec-14-enyl)resorcinol and demethylbelamcandaquinone B), gallic acid, myricetin and rutin. Of those, only gallic acid was identified in LPva water extract. Several reports have concluded that gallic acid, myricetin and rutin provide protective effects towards myocardial infarction in rats [37,38,39]. Stasiuk and Kozubek [11] have stated that alkylresorcinols prevent oxidation of fatty acids, triglycerides and phospholipids in liposomal membrane, inhibit oxidation of human low density lipoprotein *in vitro* as well as modulate activities of lipoxygenases and cyclooxygenases.

It was also reported that long-chain alkylresorcinols caused a decrease in acethylcholinesterase activity in the erythrocyte membrane while stimulating activity of Ca^2+^-dependent ATPase. All these facts may support the suggestion that the major components of LPva extracts, alkylresorcinols, (5-(*Z*-nonadec-14-enyl)resorcinol and demethylbelamcandaquinone B), gallic acid, myricetin and rutin can be used as bioactive markers of LPva standardized extracts and contributed to the cardioprotective effects of LPva against isoproterenol-induced MI rats.

Our findings have concluded that pretreatment with LPva offered dose-dependent cardioprotective effects towards isoproterenol-induced myocardial infarction in rats. Our study also demonstrates that the effects are possibly derived from its antioxidant capacity, which is directly reduced the oxidative stress during the MI and augmented the myocardial antioxidant enzyme levels while enhancing the permeability and integrity of the myocardial cell membrane.
